# Low-dose Gene Therapy Reduces the Frequency of Enzyme Replacement Therapy in a Mouse Model of Lysosomal Storage Disease

**DOI:** 10.1038/mt.2016.181

**Published:** 2016-10-25

**Authors:** Marialuisa Alliegro, Rita Ferla, Edoardo Nusco, Chiara De Leonibus, Carmine Settembre, Alberto Auricchio

**Affiliations:** 1 Telethon Institute of Genetics and Medicine (TIGEM), Pozzuoli, Naples, Italy; 2 Medical Genetics, Department of Translational Medicine (DISMET), “Federico II” University, Naples, Italy

## Abstract

Enzyme replacement therapy (ERT) is the standard of care for several lysosomal storage diseases (LSDs). ERT, however, requires multiple and costly administrations and has limited efficacy. We recently showed that a single high dose administration of adeno-associated viral vector serotype 8 (AAV2/8) is at least as effective as weekly ERT in a mouse model of mucopolysaccharidosis type VI (MPS VI). However, systemic administration of high doses of AAV might result in both cell-mediated immune responses and insertional mutagenesis. Here we evaluated whether the combination of low doses of AAV2/8 with a less frequent (monthly) than canonical (weekly) ERT schedule may be as effective as the single treatments at high doses or frequent regimen. A greater reduction of both urinary glycosaminoglycans, considered a sensitive biomarker of therapeutic efficacy, and storage in the myocardium and heart valves was observed in mice receiving the combined than the single therapies. Importantly, these levels of correction were similar to those we obtained in a previous study following either high doses of AAV2/8 or weekly ERT. Our data show that low-dose gene therapy can be used as a means to rarify ERT administration, thus reducing both the risks and costs associated with either therapies.

## Introduction

Lysosomal storage diseases (LSDs) include more than 40 distinct inherited metabolic diseases as autosomal or X-linked recessive. The majority of LSDs are caused by deficient activity of specific lysosomal hydrolases and the progressive accumulation of their substrate(s), which ultimately leads to multisystem cellular and organ dysfunction.^1^ Lysosomal enzymes are targeted to the lysosomes following binding to the mannose 6-phosphate receptor, but can also be secreted. Extracellular phosphorylated or non-phosphorylated enzyme is taken up by the distal cells via either the mannose 6-phosphate receptors or the mannose receptor located on the plasma membrane, and then trafficked to the lysosome.^2^ This is the basis for cross-correction of deficient cells through enzyme replacement therapy (ERT), which is currently the standard of care for several LSDs.^3^

Despite its ability to ameliorate patient outcomes and slow disease progression, ERT has limited efficacy on some LSDs features, such as those related to bone, brain, cartilage, heart, and eye likely because of the poor biodistribution of recombinant enzymes through the bloodstream to these regions.^3,4^ In addition, the requirement of weekly or biweekly intravenous (i.v.) infusions, which is due to the short plasma half-life of recombinant enzymes,^5,6^ carries a risk of immune-mediated allergic reactions^7^ and often requires a central venous access, resulting in a low quality of life for the patients. Finally, ERTs are extremely expensive and this represents a barrier for their widespread use in less developed countries.^4,8^ Therefore, there is high need to develop new therapeutic strategies with comparable or better efficacy than ERT, but without the inconvenience of multiple infusions associated to ERT.

Gene therapy is emerging as a successful strategy for the treatment of inherited diseases, including LSDs.^9,10,11^ Vectors based on adeno-associated viruses (AAVs) are the most frequently used for *in vivo* applications of gene therapy, because of their safety profile, wide tropism and ability to provide long-term transgene expression.^12^

AAV-mediated gene therapy has been successful in both small and large animal models of LSDs, including Pompe disease, Fabry disease, and mucopolysaccharidoses (MPS).^13,14,15,16,17,18,19,20,21,22,23,24^ In particular, AAV vectors serotype 8 (AAV2/8) are being explored to convert the liver into a factory organ for the systemic release of therapeutic proteins. A recent clinical trial using i.v. administrations of AAV2/8 in patients with hemophilia B proved the safety and efficacy of AAV2/8 liver gene transfer,^25^ resulting in long-term expression of factor IX (FIX) at therapeutic levels.^26,27^

We used a similar approach in animal models of mucopolysaccharidosis type VI (MPS VI; OMIM #253200), a rare LSD caused by deficiency of arylsulfatase B (ARSB), which leads to the lysosomal accumulation and urinary excretion of elevated amounts of the glycosaminoglycan (GAG) dermatan sulfate.^28^ We demonstrated that a single systemic administration of AAV2/8 encoding ARSB is able to convert the liver into a stable and long-term source of systemic ARSB. Since the primary involvement of the central nervous system (CNS) is absent in both MPS VI patients and animal models, this results in long-term therapeutic efficacy in both rodent and feline models of MPS VI.^13,14,15,16^

Levels of serum ARSB activity falling within the normal range were detected in MPS VI cats up to 6 years postinjection (Ferla R., Haskins M., and Auricchio, A, data not shown). In addition, gene therapy at the dose of 2 × 10^12^ gc/kg was at least as effective as weekly infusions of recombinant human ARSB (rhARSB) in a mouse model of MPS VI, suggesting that it may be preferable to ERT as it potentially requires a single i.v. administration.^15^ If this translates to humans, gene therapy could significantly improve the quality of life of MPS VI patients.

However, gene therapy at high doses may have some limitations. In the AAV2/8 clinical trial for hemophilia B, an increase in liver enzymes was observed in subjects receiving the high dose of vector (2 × 10^12^ gc/kg), likely due to cell-mediated immune responses to AAV8, which lead to the elimination of transduced hepatocytes. This increase in liver transaminases was successfully controlled with a short course of glucocorticoids,^26,27^ however either close monitoring of liver enzymes or prophylactic oral corticosteroids are required to avoid loss of transgene expression.

Additionally, issues have recently arisen concerning the risk of AAV vectors to cause insertional mutagenesis when administered at high doses. Integration of vector DNA at highly transcribed loci has been associated with hepatocellular carcinoma (HCC) in mice.^29,30,31,32,33^ More importantly, Chandler and colleagues have demonstrated that HCC incidence is AAV dose-dependent in newborn-injected mice.^29^ Furthermore, a recent study showed insertional mutagenesis due to wild-type AAV2 in human HCC,^31^ although a direct role of wild-type AAV2 in human liver tumorigenesis needs to be further investigated.

Combined therapies have been evaluated to target the pathogenic mechanisms that are at the basis of LSDs. Although LSDs are monogenic diseases, multiple secondary mechanisms and effects play a role in their pathogenesis, and each of these is a potential therapeutic target. Thus, given the complex nature of these diseases, combining therapies is a reasonable approach to target different pathways or tissues.^34^ Combination of ERT and bone marrow transplantation was tested in both MPS VII and II mouse models with additive effects on therapeutic outcome.^35,36^ Synergy between ERT and pharmacological chaperones was reported in Pompe and Fabry disease fibroblasts.^37,38^ Also, a combined regimen of ERT and hematopoietic stem cell transplantation has led to reduced morbidity and mortality in patients with either MPS I or VI.^39,40^ Dramatic synergy was observed when CNS-directed AAV2/5-mediated gene therapy was combined with bone marrow transplantation in murine models of infantile neuronal ceroid lipofuscinosis and globoid-cell leukodystrophy.^41,42,43^ Recently, the combination of CNS-directed gene therapy, substrate reduction therapy, and bone marrow transplantation resulted in an unprecedented phenotype improvement in globoid-cell leukodystrophy mice.^44^ The combination of antiinflammatory drugs with CNS-directed gene therapy improves the therapeutic outcome in a mouse model of infantile neuronal ceroid lipofuscinosis.^45^ Similarly, combined ERT and TNF-alpha have been demonstrated to improve mobility and motor activity compared with the corresponding single therapies in MPS VI rats.^46^ Finally, CNS-directed and systemic gene therapy approaches have been combined to target different tissues involved in LSDs with synergistic or additive effects.^47,48^ These studies provide the proof-of principle that combinatorial therapies are effective in LSDs.

To reduce the safety concerns and costs associated with either gene therapy or ERT at high doses or frequent regimen, here we evaluated if the combination of low doses of AAV2/8 (< 2 × 10^12^ gc/kg) with a less frequent (herein defined as monthly) than canonical ERT schedule may be as effective as the single treatments at high doses or weekly regimen, respectively.

## Results

### Increased serum ARSB levels in MPS VI transgenic mice treated with combined monthly ERT and gene therapy

MPS VI mice received at postnatal day 30 (p30) a single i.v. administration of either 2 × 10^11^ or 6 × 10^11^ gc/kg of AAV2/8.TBG.*hARSB*, which encodes human ARSB (hARSB) under the control of the liver-specific thyroxine-binding globulin (TBG) promoter, and/or monthly i.v. injections of 1 mg/kg rhARSB (Naglazyme, BioMarin Europe, London, UK), which is the dose currently used in MPS VI patients management.^6,7,49,50,51,52^

As control, MPS VI mice were either left untreated or received a combination of monthly administrations of ERT and a single injection of the control AAV2/8.TBG.*eGFP* vector, which encodes the enhanced green fluorescence protein (eGFP) under the control of the TBG promoter.

Serum ARSB was undetectable in affected control (AF) mice (**Table 1**).

**Figure 1:**
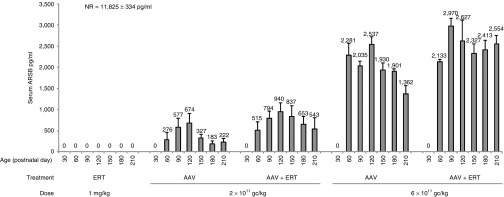
**Serum ARSB levels in mice receiving low doses of gene therapy and/or monthly ERT**. Serum ARSB (pg/ml) was monitored up to 210 days of age. Serum samples were collected monthly and before ERT administration in mice receiving ERT with or without gene therapy. Each bar represents the mean ± SE of serum ARSB levels and the corresponding value is indicated above each bar. Serum ARSB levels were undetectable in affected controls (data not shown). Values of serum ARSB levels (mean ± SE) in normal mice (NR) are shown in the figure. Number (*n*) of animals is: NR, *n* = 23 at postnatal day 30 and 150, *n* = 24 at postnatal day 60, 90, and 120, *n* = 22 at postnatal day 180 and *n* = 16 at postnatal day 210; ERT, *n* = 4; AAV 2 × 10^11^ gc/kg, *n* = 5; AAV 2 × 10^11^ + ERT, *n* = 7 except for postnatal day 210 (*n* = 4); AAV 6 × 10^11^ gc/kg, *n* = 4; AAV 6 × 10^11^ gc/kg + ERT, *n* = 5 except for postnatal day 210 (*n* = 3). The lower number of values in the later than earlier time points is due to animal sacrifice, which varied between days 180 and 210 of age. Statistical comparisons were made using the one-way analysis of variance (ANOVA) and the Tukey post hoc test. The exact *P-*values obtained are indicated in the Material and Methods section. Abbreviations: ARSB, arylsulfatase B; AAV, adeno-associated viral vector; ERT, enzyme replacement therapy.

MPS VI mice receiving either 2 × 10^11^ or 6 × 10^11^ gc/kg of AAV2/8.TBG.*hARSB* vector, with or without monthly ERT, showed a dose-dependent increase of serum ARSB levels, with a tendency to decrease at the end of the study (**Table 1** and **Figure 1**) and that corresponded to ~ 5 and 19% of normal (NR) levels, respectively. No significant differences in serum ARSB levels were observed in mice treated with combined gene therapy and monthly ERT compared with mice receiving only gene therapy (**Table 1** and **Figure 1**). This is expected based on the undetectable levels of serum ARSB in animals that received ERT administrations (**Figure 1**), which is in accordance with the peak-and-drop serum kinetics of rhARSB infusions.^5,6,15^

To confirm long-term transgene expression we measured ARSB enzyme activity and AAV vector genome copies (gc) in the livers from treated and control mice at the end of the study (**Table 1**). Persistence of liver transduction was confirmed by the presence of detectable AAV vector gc in mice receiving gene therapy. AAV gc were significantly lower (*P-*value < 0.01) in mice receiving 2 × 10^11^ gc/kg than in those treated with 6 × 10^11^ gc/kg of AAV2/8.TBG.*hARSB* in line with the dose of vector administered (**Table 1**).

### Increased urinary GAG reduction in mice receiving gene therapy in combination with ERT

Reduction of urinary GAGs is a sensitive and reliable biomarker of lysosomal storage clearance and therapeutic efficacy in LSDs.^7,49,50,51,52^

**Figure 2:**
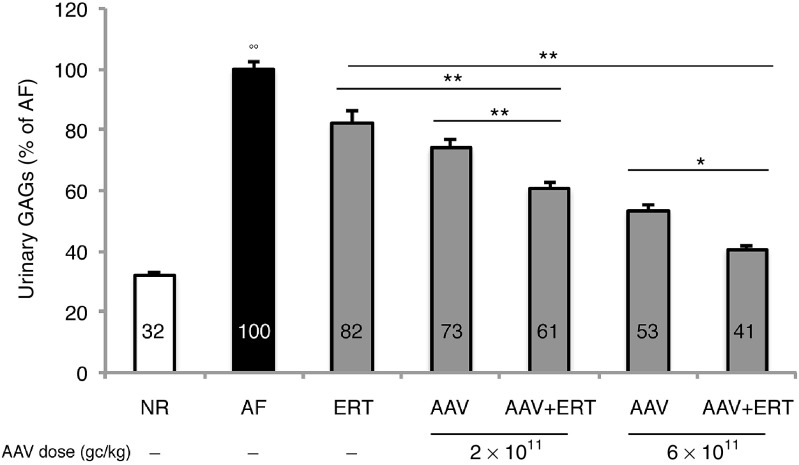
**Reduction of urinary GAGs in mice receiving low doses of gene therapy and/or monthly ERT**. Urinary GAGs were measured in treated MPS VI mice (gray bars), in normal (NR, white bars) and affected (AF, black bars) controls. Urinary GAG levels measured were averaged for all animals within the same group of treatment and the resulting value is reported as a percentage (%) of age-matched AF controls, as indicated inside each bar. Results are represented as mean ± SE. Number (*n*) of animals is: NR, *n* = 39 at postnatal day 60 and 90, *n* = 34 at postnatal day 120, *n* = 31 at postnatal day 150, *n* = 26 at postnatal day 180 and *n* = 21 at postnatal day 210; AF, *n* = 9; ERT, *n* = 5 except for postnatal day 90, 150, and 180 (*n* = 4); AAV 2 × 10^11^, *n* = 6; AAV 2 × 10^11^ + ERT, *n* = 8 except for postnatal day 210 (*n* = 5); AAV 6 × 10^11^, *n* = 5 except for postnatal day 210 (*n* = 4); AAV 6 × 10^11^ + ERT, *n* = 5 except for postnatal day 210 (*n* = 3). The lower number of values in the later than earlier time points is due to either technical challenges in the collection of samples when too numerous or to animal sacrifice, which varied between days 180 and 210 of age. Statistical comparisons were made using the one-way analysis of variance (ANOVA) and the Tukey post hoc test. The *P-*value is: *< 0.05 and **< 0.01; the *P*-value of AF versus all groups is: °° << 0.01. The exact *P*-values obtained are indicated in the Material and Methods section. Abbreviations: AAV2/8.TBG.*hARSB*, vector which encodes human ARSB; AAV, adeno-associated viral vector; ARSB, arylsulfatase B;; ERT, monthly ERT; GAGs, glycosaminoglycans; hARSB, human ARSB; MPS, mucopolysaccharidoses; TBG, thyroxine-binding globulin.

Urinary GAGs were measured monthly in MPS VI-treated mice as well as in age-matched NR and AF controls, from p60, *i.e.*, 1 month after the start of treatment. Urinary GAG levels measured at each time point were averaged for each group and the resulting value was reported as a percentage (%) of age-matched AF controls (**Figure 2** and **Supplementary Figure S1**).

Overall, urinary GAGs significantly decreased compared with AF controls in all groups of treatment (**Figure 2**). GAGs reduction in urine was observed starting from 1 month following the beginning of treatment and was stably maintained up to the end of the study for all groups (see **Supplementary Figure S1**). Specifically, only a slight reduction was observed in mice treated with monthly ERT, while a significant dose-dependent response was found in mice receiving gene therapy either alone (*P-* value AAV 2 × 10^11^ versus AAV 6 × 10^11^: << 0.01) or in combination with ERT (*P-*value AAV 2 × 10^11^ + ERT versus AAV 6 × 10^11^+ ERT: << 0.01).

More importantly, urinary GAGs decreased more in mice receiving the combined therapy than in those receiving the corresponding single treatments. Indeed, urinary GAGs were significantly lower (61% of AF) in mice treated with both 2 × 10^11^ gc/kg of AAV and ERT than in mice treated with either monthly ERT (82% of AF, *P-*value: << 0.01) or 2 × 10^11^ gc/kg of AAV (73% of AF, *P-*value: << 0.01). Likewise, a greater reduction of urinary GAGs was observed in mice receiving both 6 × 10^11^ gc/kg of AAV and ERT (41% of AF) compared with mice treated with either ERT (*P-*value: << 0.01) or gene therapy at the same dose (53% of AF, *P*-value: << 0.01) (**Figure 2**).

Specifically, the combination of gene therapy and ERT had an additive effect on the reduction of urinary GAGs.

### Amelioration of biochemical, visceral, and cardiac abnormalities in MPS VI transgenic mice treated with combined monthly ERT and gene therapy

ARSB activity and GAG levels were measured in the liver, kidney, and spleen of MPS VI-treated and control mice (**Table 1**). ARSB activity was undetectable in tissues of AF controls.

MPS VI mice receiving ERT (with or without gene therapy) were sacrificed 1 month after the last injection of rhARSB to measure the residual tissue enzymatic activity. Although ARSB activity was almost undetectable in the serum of mice treated with ERT alone, we found ARSB activity in tissues up to 1 month after injection (**Table 1**), although at levels lower than those previously measured in mice receiving weekly ERT.^15^

Increased ARSB activity was observed in the liver of all treated mice; detectable activity was variably observed in the spleen and kidney of treated mice, although at levels lower than those measured in the liver (**Table 1**). Specifically, a statistically significant increase in ARSB activity compared with AF was observed in all treated groups but the one that received monthly ERT. In addition, the levels of ARSB activity in liver correlate with the dose of vector administered (*P-*value < 0.01).

Beta-glucuronidase (GUSB) activity has been found to be secondarily increased in tissues from MPS VI cats as result of ARSB deficiency.^53^ As further therapeutic endpoint, GUSB activity was thus evaluated in tissues of MPS VI mice treated with either the combination of 6 × 10^11^ gc/kg of AAV and ERT or single therapies, and in NR and AF controls. GUSB activity was significantly increased in liver and kidney but not in spleen of MPS VI mice compared with NR (see **Supplementary Figure S2a,b**). While GUSB activity was completely normalized in liver regardless of the treatment, only mice receiving the combined therapy showed normalized levels of GUSB activity in kidney, although none of the groups of treatment was statistically different than NR controls (see **Supplementary Figure S2a,b**). The fact that liver is both the site of ARSB production following gene transfer and a more vascularized organ compared with kidney may explain why each treatment was able to restore normal GUSB activity in liver, while only the combined therapy was in kidney.

**Figure 3:**
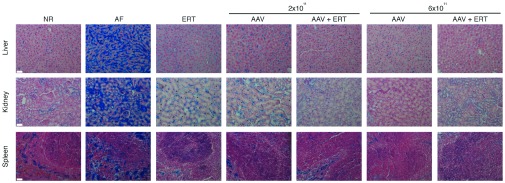
**Alcian blue staining in the liver, kidney and spleen of mice receiving low doses of gene therapy and/or monthly ERT**. Reduction of GAGs storage in the liver, kidney, and spleen was also evaluated by Alcian blue staining of histological sections obtained from MPS VI mice receiving low doses of AAV and/or monthly ERT and from normal (NR) and affected (AF) mice. All MPS VI treated mice that were sacrificed between days 180 and 210 of age were included in the histological analysis. Representative images are shown. Scale bar is 40 µm (magnification is 20×). AAV, adeno-associated viral vector; ERT, monthly ERT; GAGs, glycosaminoglycans; MPS, mucopolysaccharidoses.

More importantly, a statistically significant reduction of GAGs storage was observed in these organs, regardless of treatment and ARSB levels (**Table 1**), similarly to what was observed in mice administered with high doses of AAV2/8 or weekly ERT.^15^ This supports previous data indicating that low enzymatic levels are sufficient to improve the MPS VI visceral phenotype.^13,14,15^ Specifically, tissue GAGs in all groups were not statistically different than in normal controls, except for the spleen and kidney of mice receiving 2 × 10^11^ gc/kg (*P-*value < 0.05). In particular, GAGs were completely normalized in the visceral organs of all mice receiving 2 × 10^11^ gc/kg and ERT or gene therapy at the dose of 6 × 10^11^ gc/kg with or without ERT. Alcian blue staining of tissues confirmed the reduction of lysosomal GAG storage in the liver, kidney, and spleen of MPS VI-treated mice (**Figure 3**).

Cardiomyopathy and heart valve involvement are serious clinical complications of MPS VI that often negatively affect its prognosis.^54^ The MPS VI mouse mimics the human MPS VI cardiac phenotype.^15,55^

**Figure 4:**
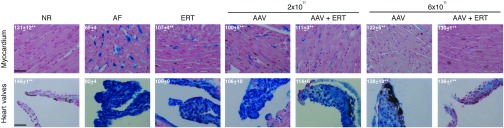
**Reduction of GAG storage in the heart valves and myocardium of mice receiving low doses of gene therapy and/or monthly ERT.** Reduction of GAGs storage in the heart valves and myocardium was evaluated by Alcian blue staining of histological sections obtained from MPS VI mice receiving AAV.TBG.*hARSB* (AAV) and/or monthly ERT (ERT) and from normal (NR) and affected (AF) controls. All MPS VI treated mice that were sacrificed between days 180 and 210 of age were included in the histological analysis. Representative images are shown. The scale bar is 40 μm (magnification is 40×). Alcian blue staining was quantified as a measure of GAGs storage in heart valves and myocardium. Specifically, Alcian Blue was quantified using the Image J software by measuring RGB intensity on images of histological sections. Results are reported inside each representative image as mean ± SE. Number (*n*) of animals is: NR, *n* = 3, AF, *N* = 3, ERT, *N* = 3, AAV 2 × 10^11^, *n* = 4; AAV 2 × 10^11^ + ERT, *n* = 5, AAV 6 × 10^11^, *n* = 3; AAV 6 × 10^11^ + ERT, *n* = 3. Statistical comparisons were made using the one-way analysis of variance (ANOVA) and the Tukey post hoc test. The *P-*value versus AF is: ** < 0.01.The exact *P-*values obtained are indicated in the Material and Methods section. AAV, adeno-associated viral vector; ARSB, arylsulfatase B; GAGs, glycosaminoglycans; hARSB, human ARSB; MPS, mucopolysaccharidoses; TBG, thyroxine-binding globulin.

We performed Alcian blue staining on heart histological sections from treated and control mice (**Figure 4**) and found a marked reduction of GAG levels in the myocardium of MPS VI mice, with the exception of those receiving either ERT or 2 × 10^11^ gc/kg of AAV, where only a slight reduction was observed. In particular, GAG levels in mice receiving 6 × 10^11^ gc/kg of AAV were comparable with those of normal controls. The Alcian blue staining quantification in heart valves and myocardium shows just a slight reduction in mice treated with 2 × 10^11^ gc/kg of AAV and/or monthly ERT. The reduction was more consistent in the cohort which received 6 × 10^11^ gc/kg of AAV, especially in combination with ERT, where GAG storage was comparable with either NR controls (**Figure 4**) or MPS VI mice which received either high doses of AAV, *i.e.*, 2 × 10^12^ gc/kg, or weekly ERT.^15^ For each treatment group, similar RGB values were observed in both myocardium and heart valves despite the different extent of blue areas in these tissues, because the quantification was performed by selecting blue areas which are homogenously present in heart valves while interspersed with nonblue fibers in myocardium.

## Discussion

In this study we show that similar efficacy to gene therapy at high doses (2 × 10^12^ gc/kg) or frequent regimen (weekly,^15^) of ERT can be obtained by combining low doses of AAV2/8 (< 2 × 10^12^ gc/kg) with rarified (monthly) ERT. In particular, this was demonstrated by a great reduction of both urinary GAGs and storage in myocardium and heart valves observed in mice receiving combined monthly ERT and gene therapy at dose 6 × 10^11^ gc/kg. These levels of correction were similar to normal controls as previously observed in mice administered with single treatments at high doses of vector or weekly schedule of ERT.^15^

The therapeutic impact on skeletal disease was not evaluated in this study. Indeed, no improvement in bone abnormalities was observed in adult mice (p30) treated with high doses of vector or frequent regiment of ERT.^15^ Also, no response was observed in mice following neonatal gene transfer.^15^ In MPS VI transgenic mice treated at birth, we found that serum ARSB peaks to supraphysiological levels and then rapidly drops to below normal levels few weeks after injection (unpublished data), because of vector dilution due to hepatocytes proliferation.^56,57^ This may explain the absence of bone responses after neonatal gene transfer in MPS VI mice.

Neonatal ERT has not been tested in MPS VI mice. However, MPS VI cats treated at birth with ERT showed only a slight amelioration in physical appearance.^58^ In our previous study we also showed that the improvement in MPS VI mice behavior provided by gene therapy over ERT was mostly due to the reduction in stress associated with repeated ERT systemic administrations.^15^ This, and the fact that the behavioral improvement albeit significant was modest, has led us to exclude this endpoint from the comparative assessment of multiple therapeutic regimens as those presented here, where we have selected only those endpoints that better responded to either gene therapy or ERT.

We demonstrate that gene therapy may be regarded as a means to decrease the frequency of ERT infusions. While gene therapy could provide baseline enzyme levels to taper GAG levels, the high intracellular levels of therapeutic enzyme achieved with ERT can be used only occasionally to help clear tissues from any GAGs storage in excess. The use of a rarified ERT schedule should lead to several important advantages, including reduction of both allergic reaction associated with the frequent infusion of recombinant enzyme and the costs of ERT, that range between euro 150,000 and 450,000 per patient/year^8^ (depending on the patient weight) in the case of MPS VI. The high costs may limit the access to the therapy to patients living in less developed countries and where therapies are not supported by the public health system.^4,8^ This scenario may change if a single administration of low dose gene therapy allows rarifying the ERT schedule.

Recently, the universal safety of AAV was questioned by studies, which described the development of HCC in mice after systemic delivery of an AAV gene therapy vector.^29,30^ However, the potential oncogenic risk seems to be influenced by several factors, such as the age of administration, the control elements used to drive transgene expression and, importantly, the vector dose administered.^29,30,32,33^ Indeed, Chandler's study showed that reducing the dose of AAV8 decreases the incidence of HCC to control levels following neonatal gene transfer, regardless of the enhancer promoter used to drive transgene expression.^29^ Thus, reducing the doses of AAV systemic administrations should reduce the risk of genotoxicity.^29^

In addition, the hepatocellular toxicity due to cell-mediated immune responses to AAV8 observed at the 2 × 10^12^ gc/kg dose of AAV2/8 in hemophilia B patients was not observed in any of the patients who received 6 × 10^11^ gc/kg of AAV2/8,^26,27^ the dose that we show here to be as effective as 2 × 10^12^ gc/kg^15^ if combined with monthly ERT.

An additional potential advantage of combining gene and protein delivery is that liver-directed gene therapy has been demonstrated to either prevent the generation of humoral immunity to the transgene product in several models of LSDs^59^ or to eradicate it, if already present.^60,61,62^ Notably, immune-modulatory gene therapy with a subtherapeutic dose of vector was shown to enhance the efficacy of ERT in murine Pompe disease by preventing the generation of humoral immunity to recombinant alfa-glucosidase.^20,63^ Therefore, gene therapy may also positively impact on ERT therapeutic efficacy and safety by avoiding the generation of inhibitors to therapeutic proteins, which is a limit to the successful treatment of several inherited diseases.

Last but not least, this study helps managing patients with LSDs for which ERT is available and who are enrolled in gene therapy clinical trials. We are indeed developing a phase 1/2 study to test the efficacy of gene therapy for MPS VI (http://meusix.tigem.it). If efficacy is observed that is inferior to that observed during ERT, these patients who have received gene therapy could be put on a rarified rather than on the canonical highly frequent ERT schedule.

In this study, we show in a mouse model the therapeutic efficacy of a novel combinatorial gene therapy/ERT approach for MPS VI, and potentially other LSDs. By taking advantage of the different pharmacokinetics and dynamics of either approach, this combination has the potential to reduce the risks and costs associated with gene therapy and ERT, respectively.

## Materials and Methods

***Animal colony.*** MPS VI mice were maintained at the Cardarelli Hospital Animal House (Naples, Italy). Animals were raised in accordance with the Institutional Animal Care and Use Committee (IACUC) guidelines for the care and use of animals in research. This mouse model carries a targeted disruption of the *ARSB* locus^64^ and is made immune-tolerant to human ARSB by transgenic insertion of the C91S *hARSB* mutant, resulting in the production of inactive hARSB.^65^ Six out of 38 MPS VI mice from the same genetic background had the C91S *hARSB* transgene inserted into the ROSA26 locus,^66^ while the remaining presented random integrations of this transgene.^15^ Genotype analysis was performed by polymerase chain reaction (PCR) on genomic DNA obtained from the tail, as previously described.^15^

***Plasmid and vector production.*** The plasmid pAAV2.1.TBG.*hARSB* encoding the hARSB protein was generated, as described previously.^19^ The therapeutic AAV2/8.TBG*.hARSB* and the control AAV2/8.TBG.*eGFP* vectors were produced by the AAV Vector Core (Telethon Institute of Genetics and Medicine (TIGEM), Pozzuoli, Naples, Italy), as previously described.^14^

***Treatment administration.*** MPS VI mice were treated with gene therapy and/or ERT through i.v. retro-orbital injections, starting from p30 to avoid vector dilution due to hepatocyte proliferation^13,56,57^ and were followed up to 6–7 months of age. MPS VI mice were treated with a single injection of the therapeutic AAV2/8.TBG.*hARSB* vector at either 2 × 10^11^ or 6 × 10^11^ gc/kg and/or monthly injections of 1 mg/kg rhARSB protein (Naglazyme, BioMarin Europe, London, UK), appropriately diluted in phosphate buffered saline (PBS). As control, MPS VI mice either received monthly administrations of PBS (*n* = 1) or a single injection of the control AAV2/8.TBG.*eGFP* vector (*n* = 1) or were left untreated (*n* = 8). Both male and female mice are included in each group of treatment.

***Blood, urine, and tissue collection.*** Blood was collected each month from treated and control mice and before ERT administration in mice receiving ERT with or without gene therapy. Serum samples were collected via eye bleeding and centrifuged at 10,000×*g* in a microcentrifuge (Z 216 MK, HERMLE, Wehingen, Germany) for 10 minutes at 4ºC to obtain the serum.

Urine was also collected monthly before each ERT administration in mice receiving ERT with or without gene therapy. Specifically, mice were put in metabolic cages for 24 hours. The urine samples were briefly centrifuged to remove debris and stored at −20 °C.

Mice were sacrificed 5 or 6 months following the start of treatment and 1 month after the last ERT administration. A cardiac perfusion with PBS was performed, and the liver, kidney, spleen, and heart were collected. Tissue samples were fixed in a methacarn solution (30% chloroform, 60% methanol, and 10% acetic acid) for 24 hours or frozen in dry ice (for ARSB activity and GAG quantitative assays).

***Immune capture assay for determination of serum ARSB enzymatic activity.*** Serum ARSB activity was measured by an immune capture assay based on the use of a specific anti-hARSB polyclonal antibody (Covalab, Villeurbanne, France). Ninety-six-well plates (Nunclon, Roskilde, Denmark) were coated with 5 µg/ml in 0.1 mol/l NaHCO_3_ (100 µl/well) and incubated overnight (O/N) at 4^o^C. The following day, plates were washed twice with 0.25 mol/l NaCl/0.02 mol/l Tris, pH 7, and then blocked with 1% milk in 0.25 mol/l NaCl/0.02 mol/l Tris, pH 7 (blocking solution), for 2 hours at room temperature. Plates were washed again, as described above, and then 50 µl of standard and unknown samples (diluted 1:10) were added to each well. Plates were shaken for 1 hour at room temperature and then incubated at 4°C overnight. The following day, plates were shaken for 1 hour at room temperature and then washed 2× with 0.25 mol/l NaCl/0.02 mol/l Tris, pH 7. In total, 100 µl of 5 mmol/l 4-methylumbelliferylsulfate potassium salt (4-MUS, Sigma-Aldrich, Milan, Italy) substrate was added to each well and then incubated at 37°C for 4 hours. The reaction was stopped by the addition of 100 µl/well of stop solution (glycine 0.2 mol/l). Plates were shaken for 10 minutes at room temperature and the fluorescence was read (excitation 365 nm/emission 460 nm) on a multiplate fluorimeter (TECAN Infinite F200, Männedorf, Switzerland). Serum ARSB was determined based on a rhARSB (Naglazyme, BioMarin Europe, London, UK) standard curve and expressed as pg/ml.

***AAV vector genome distribution.*** Genomic DNA was extracted from the livers using the DNeasy Blood and Tissue Extraction kit (Qiagen, Hilden, Germany). Real-time PCR analysis was performed on 100 ng of genomic DNA using a set of primers/probe (Fw 5′-TCTAGTTGCCAGCCATCTGTTGT-3′, Rev 5′-TGGGAGTGGCACCTTCCA-3′, Probe 5′-TCCCCCGTGCCTTCCTTGACC-3′) specific for the viral genome and Taq-Man universal PCR master mix (Applied Biosystems, Foster City, CA). Amplification was run on a 7300 Real-Time PCR system (Applied Biosystems) with standard cycles. All the reactions were performed in triplicate.

***Assay for ARSB and GUSB enzymatic activity evaluation in tissues.*** Tissues, *i.e.*, liver, kidney, and spleen were homogenized in water and protein concentrations were determined with the bicinchoninic acid (BCA) protein assay kit (Pierce Protein Research Products, Thermo Fisher Scientific, Rockford, IL). The ARSB assay was performed, as previously described.^67^ Briefly, 30 µg of protein was incubated with 40 µl of the fluorogenic4-methylumbelliferyl sulfate substrate (12.5 mmol/l; Sigma-Aldrich, Saint Louis, MO) for 3 hours at 37°C in the presence of 40 µl silver nitrate (0.75 mmol/l; Carlo Erba, Milan, Italy), which is known to inhibit the activity of other sulfatases. The reaction was stopped by adding 200 µl of carbonate glycine stop buffer and the fluorescence of the 4-methylumbelliferone liberated was measured on a multiplate reader (TECAN Infinite F200) at 365 nm (excitation) and 460 nm (emission).

β-glucuronidase (GUSB) assay was performed, as previously described.^68^ Briefly, 200 µg of protein was incubated with 400 µl of GUS assay buffer (50 mmol/l NaPO_4_ pH 7, 5 mmol/l DTT, 1 mmol/l EDTA, 0.1% Triton X-100) and 100 µl of the fluorogenic 4-methylumbelliferyl-β-d-glucuronide (MUG) substrate (5 mmol/l; Sigma-Aldrich) for 30 minutes at 37°C. The reaction was stopped by adding 900 µl of stop buffer (0.2 mmol/l Na_2_CO_3_, pH 9.5) to 50 µl of sample. The fluorescence was measured on a multiplate reader (GloMax-Multi detection system Promega, Promega S.r.l. Milano, Italia) at 388 nm (excitation) and 480 nm (emission).

Enzyme activities were calculated with a standard curve of the fluorogenic4-methylumbelliferone product (12.5 mmol/l; Sigma-Aldrich). For tissue lysates the activity was expressed as nanomoles per milligram of protein per hour (nmol/mg/h).

***Quantitative analyses of GAG accumulation in tissues and urine.*** Urine samples were diluted 1:50 in water to measure GAG content. One hundred microliters of diluted urine or 250 µg of protein extract from the liver, spleen, and kidney was used for the GAG assay, as previously described.^69^ GAG concentrations were determined on the basis of a dermatan sulfate standard curve (Sigma-Aldrich). Tissue GAGs were expressed as micrograms of GAG per milligram of protein (µg GAG/mg protein). Urinary GAGs were normalized to creatinine content which was measured with a creatinine assay kit (Quidel, San Diego, CA). Thus, the units of urinary GAGs are micrograms per micromole of creatinine (µg GAG/µmol creatinine). Urinary GAGs were reported as percentage of AF control mice. The urinary GAG levels measured at each time point were averaged for each group.

***Alcian blue staining.*** After methacarn fixation, all the tissues (liver, spleen, kidney, and heart) were dehydrated through immersion in alcohols at increasing concentration (70, 80, 90, and 100%) and then in xylene. Tissues were embedded in paraffin and sectioned into 7-µm-thick serial sections on a microtome. Tissue sections were deparaffinized, rehydrated through immersion in alcohols at decreasing concentration (100, 95, 80, and 70%) and then water and stained with 1% Alcian blue (Sigma-Aldrich) in hydrochloric acid for 10 seconds. Counterstaining was performed for 1 minute with nuclear-fast red (Sigma-Aldrich). Alcian blue staining in heart valves and myocardium was quantified to provide a measure of GAG storage. Specifically, Alcian blue staining was quantified by measuring RGB intensity on histological section using the Image J software. RGB may assume integer values from 0 to 255. The more intense is the Alcian blue staining, the lower is the RBG value. Four different areas were randomly selected in each valve. As far as myocardium, five areas corresponding to Alcian blue spots were randomly selected per each section. Where Alcian blue spots were not present, as in NR and some treated mice, five equivalent areas were randomly selected. RGB was measured per each area and then averaged for each animal and each group of animals, as reported in **Figure 4**.

***Statistical analyses.*** All results are expressed as mean ± standard error (SE). Statistical comparisons were made using either *t*-test or one-way analysis of variance (ANOVA); the Tukey post hoc test was used. Statistical significance was considered if *P* < 0.05. The exact *P-*value for each comparison follows (**Table 1**): Serum ARSB: The ANOVA *P-*value is 2.00 e^−16^; the *P-*value of NR versus AF is: 4.88e^−13^; the *P-*value of ERT versus AF is: 1.00; the *P-*value of AAV 2 × 10^11^ versus AF is: 0.99; the *P-*value of AAV 2 × 10^11^ + ERT versus AF is: 0.83; the *P-*value of AAV 6 × 10^11^ versus AF is: 0.02; the *P-*value of AAV 6 × 10^11^+ ERT versus AF is: 6.90e^−4^.

Liver genome copies: The ANOVA *P-*value is 4.85e^−8^; the *P-*value of AAV 2 × 10^11^ versus AAV 6 × 10^11^ is: 2.20e^−6^; the *P-*value of AAV 2 × 10^11^ + ERT versus AAV 6 × 10^11^ + ERT is: 3.70e^−6^; the *P-*value of AAV 2 × 10^11^ versus AAV 2 × 10^11^ + ERT is: 0.98; the *P-*value of AAV 6 × 10^11^ versus AAV 6 × 10^11^ + ERT is: 0.99.

Liver ARSB activity: The ANOVA *P-*value is 3.75e^−9^; the *P-*value of ERT *versus* AF is: 0.58; the *P-*value of AAV 2 × 10^11^ versus AF is: 0.03; the *P-*value of AAV 2 × 10^11^ + ERT versus AF is: 1.00e^−3^; the *P-*value of AAV 6 × 10^11^ versus AF is: 1.00e^−7^; the *P-*value of AAV 6 × 10^11^+ ERT versus AF is: < 3.75e^−9^; the *P-*value of AAV 2 × 10^11^ versus AAV 6 × 10^11^ is: 1.00e^−3^; the *P-*value of AAV 2 × 10^11^ + ERT versus AAV 6 × 10^11^ + ERT is: 6.00e^−3^; the *P-*value of NR versus AF is 2.17e^−8^.

Liver GAGs: The ANOVA *P-*value is 7.93e^−21^; the *P-*value of NR versus AF is < 7.93e^−21^; the *P-*value of ERT versus AF is: < 7.93e^−21^; the *P-*value of AAV 2 × 10^11^ versus AF is: < 7.93e^−21^; the *P-*value of AAV 2 × 10^11^ + ERT versus AF is: < 7.93e^−21^; the *P-*value of AAV 6 × 10^11^ versus AF is: < 7.93e^−21^; the *P-*value of AAV 6 × 10^11^+ ERT versus AF is: < 7.93e^−21^; the *P-*value of ERT versus NR is: 0.99; the *P-*value of AAV 2 × 10^11^ versus NR is: 0.99; the *P-*value of AAV 2 × 10^11^ + ERT versus NR is: 0.99; the *P-*value of AAV 6 × 10^11^ versus NR is: 0.99; the *P-*value of AAV 6 × 10^11^+ ERT versus NR is: 0.99.

Kidney ARSB activity: The ANOVA *P-*value is 1.28e^−5^; the *P-*value of ERT versus AF is: 0.22; the *P-*value of AAV 2 × 10^11^ versus AF is: 2.00e^−3^; the *P-*value of AAV 2 × 10^11^ + ERT versus AF is: 8.20e^−5^; the *P-*value of AAV 6 × 10^11^ versus AF is: 8.30e^−5^; the *P-*value of AAV 6 × 10^11^+ ERT versus AF is: 1.00e^−3^; the *P-*value of NR versus AF is 1.80e^−8^.

Kidney GAGs: The ANOVA *P-*value is 1.16e^−15^; the *P-*value of NR versus AF is < 1.16e^−15^; the *P-*value of ERT versus AF is: 1.00e^−7^; the *P-*value of AAV 2 × 10^11^ versus AF is: 4.00e^−7^; the *P-*value of AAV 2 × 10^11^ + ERT versus AF is: < 1.16e^−15^; the *P-*value of AAV 6 × 10^11^ versus AF is: < 1.16e^−15^; the *P-*value of AAV 6 × 10^11^+ ERT versus AF is: < 1.16e^−15^; the *P-*value of ERT versus NR is: 0.42; the *P-*value of AAV 2 × 10^11^ versus NR is: 0.03; the *P-*value of AAV 2 × 10^11^ + ERT versus NR is: 0.99; the *P-*value of AAV 6 × 10^11^ versus NR is: 0.99; the *P-*value of AAV 6 × 10^11^+ ERT versus NR is: 0.99.

Spleen ARSB activity: The ANOVA *P-*value is 5.73e^−6^; the *P-*value of ERT versus AF is: 0.20; the *P-*value of AAV 2 × 10^11^ versus AF is: 7.00e^−3^; the *P-*value of AAV 2 × 10^11^ + ERT versus AF is: 1.12e^−4^; the *P-*value of AAV 6 × 10^11^ versus AF is: 2.46e^−4^; the *P-*value of AAV 6 × 10^11^+ ERT versus AF is: 2.46e^−5^; the *P-*value of NR versus AF is 4.28e^−9^.

Spleen GAGs: The ANOVA *P-*value is 1.49e^−18^; the *P-*value of NR versus AF is < 1.49e^−18^; the *P-*value of ERT versus AF is: 1.08e^−11^; the *P-*value of AAV 2 × 10^11^ versus AF is: 1.67e^−10^; the *P-*value of AAV 2 × 10^11^ + ERT versus AF is: < 1.49e^−18^; the *P-*value of AAV 6 × 10^11^ versus AF is: < 1.49e^−18^; the *P-*value of AAV 6 × 10^11^+ ERT versus AF is: < 1.49e^−18^; the *P-*value of ERT versus NR is: 0.66; the *P-*value of AAV 2 × 10^11^ versus NR is: 0.04; the *P-*value of AAV 2 × 10^11^ + ERT versus NR is: 0.98; the *P-*value of AAV 6 × 10^11^ versus NR is: 0.99; the *P-*value of AAV 6 × 10^11^+ ERT versus NR is: 0.99.

Figure 1. Post-natal day 30: the ANOVA *p* value is 6.25e^-12^; the *p* value of NR vs AF is 3.32e^-7^; the *p* value of ERT vs AF is: 1.00; the* p *value of AAV 2x10^11^ vs AF is: 1.00; the* p *value of AAV 2x10^11^ + ERT vs AF is: 1.00; the *p* value of AAV 6x10^11^ vs AF is: 1.00; the* p *value of AAV 6x10^11^+ ERT vs AF is: 1.00; post-natal day 60: the ANOVA *p* value is 1.09e^-14^; the *p* value of NR vs AF is: 7.85e^-12^; the* p *value of ERT vs AF is: 1.00; the *p* value of AAV 2x10^11^ vs AF is: 0.99; the* p *value of AAV 2x10^11^ + ERT vs AF is: 0.99; the *p* value of AAV 6x10^11^ vs AF is: 0.73; the* p *value of AAV 6x10^11^+ ERT vs AF is: 0.72; post-natal day 90: the ANOVA* p *value is 4.03e^-17^; the* p *value of NR vs AF is: < 4.03e^-17^; the *p* value of ERT vs AF is: 1.00; the *p* value of AAV 2x10^11^ vs AF is: 0.99; the *p* value of AAV 2x10^11^ + ERT vs AF is: 0.99; the *p* value of AAV 6x10^11^ vs AF is: 0.91; the* p *value of AAV 6x10^11^+ ERT vs AF is: 0.56; post-natal day 120: the ANOVA *p* value is 2.45e^-16^; the *p* value of NR vs AF is: < 2.45e^-16^; the* p *value of ERT vs AF is: 1.00; the *p * value of AAV 2x10^11^ vs AF is: 0.99; the* p *value of AAV 2x10^11^ + ERT vs AF is: 0.99; the *p* value of AAV 6x10^11^ vs AF is: 0.75; the* p *value of AAV 6x10^11^+ ERT vs AF is: 0.64; post-natal day 150: the ANOVA *p* value is 5.67e^-21^; the* p *value of NR vs AF is: < 5.67e^-21^; the* p *value of ERT vs AF is: 1.00; the *p* value of AAV 2x10^11^ vs AF is: 0.99; the* p *value of AAV 2x10^11^ + ERT vs AF is: 0.99; the *p* value of AAV 6x10^11^ vs AF is: 0.78; the* p *value of AAV 6x10^11^+ ERT vs AF is: 0.51; post-natal day 180: the ANOVA *p* value is 5.06e^-21^; the *p* value of NR vs AF is: < 5.06e^-21^; the *p* value of ERT vs AF is: 1.00; the *p* value of AAV 2x10^11^ vs AF is: 0.99; the *p* value of AAV 2x10^11^ + ERT vs AF is: 0.99; the* p *value of AAV 6x10^11^ vs AF is: 0.79; the* p *value of AAV 6x10^11^+ ERT vs AF is: 0.47; post-natal day 210: the ANOVA *p* value is 7.75e^-15^; the *p* value of NR vs AF is: 5.16e^-12^; the *p* value of ERT vs AF is: 1.00; the *p* value of AAV 2x10^11^ vs AF is: 0.99; the* p *value of AAV 2x10^11^ + ERT vs AF is: 0.99; the *p* value of AAV 6x10^11^ vs AF is: 0.97; the *p* value of AAV 6x10^11^+ ERT vs AF is: 0.63.

Figure 2. The ANOVA* p *value is < 2.00 e^-16^; the *p* value of NR vs AF is < 2.00e^-16^; the *p* value of ERT vs AF is: 6.01e^-5^; the *p* value of AAV 2x10^11^ vs AF is: < 2.00e^-16^; the* p *value of AAV 2x10^11^ + ERT vs AF is: < 2.00e^-16^; the* p *value of AAV 6x10^11^ vs AF is: < 2.00e^-16^; the *p* value of AAV 6x10^11^+ ERT vs AF is: < 2.00e^-16^; the *p* value of ERT vs NR is: < 2.00e^-16^; the *p* value of AAV 2x10^11^ vs NR is: < 2.00e^-16^; the *p* value of AAV 2x10^1-1^ + ERT vs NR is: < 2.00e^-16^; the *p* value of AAV 6x10^11^ vs NR is: 4.90e^-10^; the* p *value of AAV 6x10^11^+ ERT vs NR is: 0.08; the* p *value of AAV 2x10^11^ vs AAV 6x10^11^ is: 4.20e^-6^; the *p* value of AAV 2x10^11^ + ERT vs AAV 6x10^11^ + ERT is: 2.86e^-6^; the *p* value of AAV 2x10^11^ + ERT vs ERT is: 3.81e^-7^; the *p* value of AAV 2x10^11^ + ERT vs AAV 2x10^11^ is: 3.57e^-3^; the *p* value of AAV 6x10^11^ + ERT vs ERT is: < 2.00e^-16^; the *p* value of AAV 6x10^11^ + ERT vs AAV 6x10^11^ is: 0.04.

Figure 4. RGB quantification in heart valves. The ANOVA* p *value is 4.08e^-4^; the *p* value of NR vs AF is 3.24e^-4^; the* p *value of ERT vs AF is: 0.24; the *p* value of AAV 2x10^11^ vs AF is: 0.30; the* p *value of AAV 2x10^11^ + ERT vs AF is: 0.07; the *p* value of AAV 6x10^11^ vs AF is: 9.93e^-3^; the *p* value of AAV 6x10^11^+ ERT vs AF is: 2.25e^-3^; the *p* value of ERT vs NR is: 0.04; the *p* value of AAV 2x10^11^ vs NR is: 0.01; the* p *value of AAV 2x10^11^ + ERT vs NR is: 0.05; the *p* value of AAV 6x10^11^ vs NR is: 0.62; the *p* value of AAV 6x10^11^+ ERT vs NR is: 0.95.

***R***GB quantification in myocardium. The ANOVA *p* value is 1.16e^-6^; the *p* value of NR vs AF is < 1.16e^-6^; the* p *value of ERT vs AF is: 8.38e^-4^; the *p* value of AAV 2x10^11^ vs AF is: 2.31e^-3^; the* p *value of AAV 2x10^11^ + ERT vs AF is: 7.37e^-5^; the* p *value of AAV 6x10^11^ vs AF is: 2.29e^-5^; the* p *value of AAV 6x10^11^+ ERT vs AF is: 3.60e^-6^; the *p* value of ERT vs NR is: 0.02; the *p* value of AAV 2x10^11^ vs NR is: 1.48e^-3^; the* p *value of AAV 2x10^11^ + ERT vs NR is: 0.02; the *p* value of AAV 6x10^11^ vs NR is: 0.43; the *p* value of AAV 6x10^11^+ ERT vs NR is: 0.96.

***Supplementary Figure S1***. *Post-natal day 60*: the ANOVA* p *value is 5.08e-18; the* p *value of NR vs AF is: <5.08e-18; the* p *value of ERT vs AF is: 0.37; the* p *value of AAV 2x10^11^ vs AF is: 0.03; the* p *value of AAV 2x10^11^ + ERT vs AF is: 1.17e^-5^; the *p* value of AAV 6x10^11^ vs AF is: 1.99e^-4^; the *p* value of AAV 6x10^11^ + ERT vs AF is: 1.11e^-7^; the *p* value of ERT vs NR is: 5.19e^-8^; the* p *value of AAV 2x10^11^ vs NR is: 7.04e^-7^; the* p *value of AAV 2x10^11^ + ERT vs NR is: 5.51e^-4^; the *p* value of AAV 6x10^11^ vs NR is: 7.00e^-3^; the *p* value of AAV 6x10^11^ + ERT vs NR is: 0.76; *post-natal day 90*: the ANOVA *p* value is 5.97e^-16^; the *p* value of NR vs AF is: < 5.97e^-16^; the* p *value of ERT vs AF is: 0.99; the *p* value of AAV 2x10^11^ vs AF is: 0.02; the *p* value of AAV 2x10^11^ + ERT vs AF is: 1.37e^-4^; the *p* value of AAV 6x10^11^ vs AF is: 1.36e^-5^; the *p* value of AAV 6x10^11^+ ERT vs AF is: 2.94e^-6^; the *p* value of ERT vs NR is: 1.63e^-8^; the *p* value of AAV 2x10^11^ vs NR is: 9.76e^-5^; the *p* value of AAV 2x10^11^ + ERT vs NR is: 3.00e^-3^; the *p* value of AAV 6x10^11^ vs NR is: 0.54; the *p* value of AAV 6x10^11^+ ERT vs NR is: 0.82; *post-natal day 120*: the ANOVA *p* value is 1.33e^-20^; the *p* value of NR vs AF is: 1.46e^-12^; the* p *value of ERT vs AF is: 0.41; the *p* value of AAV 2x10^11^ vs AF is: 0.09; the *p* value of AAV 2x10^11^ + ERT vs AF is: 1.20e^-4^; the *p* value of AAV 6x10^11^ vs AF is: 1.12e^-7^; the *p* value of AAV 6x10^11^+ ERT vs AF is: 6.22e^-10^; the *p* value of ERT vs NR is: 4.18e^-10^; the *p* value of AAV 2x10^11^ vs NR is: 6.96e^-10^; the *p* value of AAV 2x10^11^ + ERT vs NR is: 1.55e^-7^; the *p* value of AAV 6x10^11^ vs NR is: 0.13; the *p* value of AAV 6x10^11^+ ERT vs NR is: 0.91; *post-natal day 150*: the ANOVA *p* value is 9.28e^-15^; the *p* value of NR vs AF is: 2.04e^-11^; the* p *value of ERT vs AF is: 0.64; the *p* value of AAV 2x10^11^ vs AF is: 4.00e^-3^; the *p* value of AAV 2x10^11^ + ERT vs AF is: 4.84e^-5^; the *p* value of AAV 6x10^11^ vs AF is: 3.47e^-6^; the *p* value of AAV 6x10^11^+ ERT vs AF is: 3.51e^-7^; the *p* value of ERT vs NR is: 2.30e^-6^; the *p* value of AAV 2x10^11^ vs NR is: 2.6e^-4^; the *p* value of AAV 2x10^11^ + ERT vs NR is: 2.00e^-3^; the* p *value of AAV 6x10^11^ vs NR is: 0.51; the *p* value of AAV 6x10^11^+ ERT vs NR is: 0.90; *post-natal day 180*: the ANOVA *p* value is 5.20e^-14^; the* p *value of NR vs AF is: 7.37e^-12^; the* p *value of ERT vs AF is: 0.09; the* p *value of AAV 2x10^11^ vs AF is: 0.08; the *p* value of AAV 2x10^11^ + ERT vs AF is: 9.99e^-5^; the* p *value of AAV 6x10^11^ vs AF is: 2.47e^-4^; the* p *value of AAV 6x10^11^+ ERT vs AF is: 9.80e^-8^; the *p* value of ERT vs NR is: 2.40e^-4^; the *p* value of AAV 2x10^11^ vs NR is: 2.68e^-6^; the *p* value of AAV 2x10^11^ + ERT vs NR is: 7.73e^-4^; the *p* value of AAV 6x10^11^ vs NR is: 0.03; the *p* value of AAV 6x10^11^+ ERT vs NR is: 0.98; *post-natal day 210*: the ANOVA *p* value is 1.84e^-13^; the *p* value of NR vs AF is: 1.72e^-12^; the *p* value of ERT vs AF is: 0.05; the *p* value of AAV 2x10^11^ vs AF is: 0.07; the* p *value of AAV 2x10^11^ + ERT vs AF is: 1.03e^-4^; the* p *value of AAV 6x10^11^ vs AF is: 1.00e^-3^; the *p* value of AAV 6x10^11^+ ERT vs AF is: 5.73e^-7^; the *p* value of ERT vs NR is: 1.01e^-6^; the* p *value of AAV 2x10^11^ vs NR is: 5.50e^-8^; the p value of AAV 2x10^11^ + ERT vs NR is: 4.00e^-3^; the *p* value of AAV 6x10^11^ vs NR is: 2.00e^-3^; the* p *value of AAV 6x10^11^+ ERT vs NR is: 0.99. 

***Supplementary Figure S2***. Liver β-glucuronidase activity (a): The ANOVA* p *value is 8.88e^-3^; the* p *value of NR vs AF is 0.05; the* p *value of ERT vs AF is 0.03; the* p *value of AAV 6x10^11^ vs AF is 0,03; the *p* value of AAV 6x10^11^ + ERT vs AF is 0.08; the* p *value of ERT vs NR is 0.99; the* p *value of AAV 6x10^11^ vs NR is 0.99; the* p *value of AAV 6x10^11^ + ERT vs NR is 0.90.

***K***idney ***β-***glucuronidase activity (b). The ANOVA* p *value is 7.16e^-7^; the *p* value of NR vs AF is 1.00e^-6^; the* p *value of ERT vs AF is 2.50e^-4^; the *p* value of AAV 6x10^11^ vs AF is 8.99e^-5^; the *p* value of AAV 6x10^11^ + ERT vs AF is 2.30e^-6^; the* p *value of ERT vs NR is 0.09; the *p* value of AAV 6x10^11^ vs NR is 0.21; the *p* value of AAV 6x10^11^ + ERT vs NR is 0.99.

**SUPPLEMENTARY MATERIAL** **Figure S1.** Reduction of urinary GAGs in mice receiving low doses of gene therapy 1and/or monthly ERT. **Figure S2.** Reduction of liver and kidney GUSB activity in mice receiving low doses of 19 gene therapy and/or monthly ERT.

## Figures and Tables

**Table 1:**
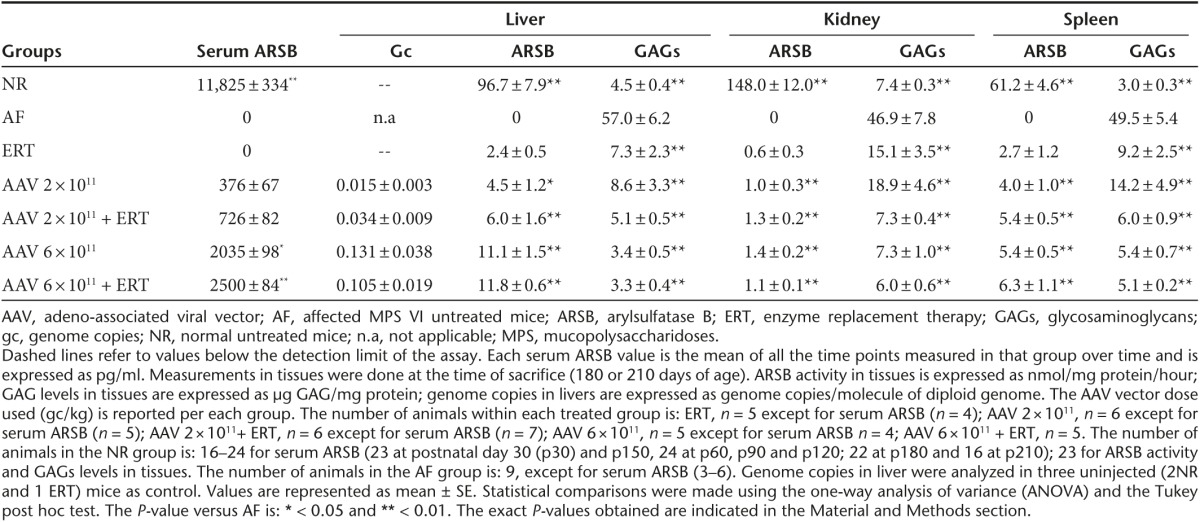
Liver vector genome copies, serum and peripheral tissue ARSB and GAGs in MPS VI mice receiving low doses of gene therapy and/or monthly ERT
