# Supplementary material for: Low-dose Gene Therapy Reduces the Frequency of Enzyme Replacement Therapy in a Mouse Model of Lysosomal Storage Disease
Source: Mol Ther. 2016 Oct 25;24(12):2054–63. doi: 10.1038/mt.2016.181 (PMC5159621; doi:10.1038/mt.2016.181)
Supplement: Supplementary Figures [file mt2016181x1.pdf]

Figure S1. Reduction of urinary GAGs in mice receiving low doses of gene therapy and/or monthly ERT.

Figure S1

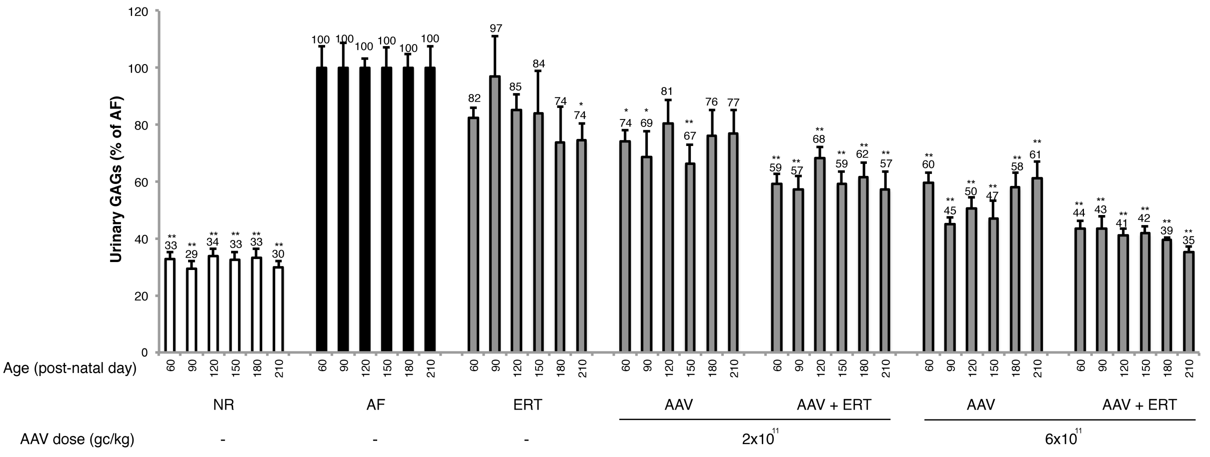

Urinary GAGs were measured in treated MPS VI mice (gray bars), in normal (NR, white bars) and in affected (AF, black bars) controls. Urinary GAG levels measured at each time point were averaged for all animals within the same group of treatment and the resulting value is reported as a percentage (%) of age-matched AF controls, as indicated above each bar. Results are represented as mean ± SE. Number (n) of animals is: NR, n=39 at post-natal day 60 and 90, n=34 at post-natal day 120, n=31 at post-natal day 150, n=27 at post-natal day 180 and n=21 at post-natal day 210; AF, n=9; ERT, n=5 except for post-natal day 90, 150 and 180 (n=4); AAV $2x10^{11}$, n=6; AAV $2x10^{11}$ + ERT, n=8 except for post-natal day 210 (n=5); AAV $6x10^{11}$, n=5 except for post-natal day 210 (n=4); AAV $6x10^{11}$ + ERT, n=5 except for post-natal day 210 (n=3). The lower number of values in the later than earlier time points is due to either technical challenges in the collection of samples when too numerous or to animal sacrifice, which varied between days 180 and 210 of age. Statistical comparisons were made using the one-way ANOVA and the Tukey post hoc test. The p value vs. AF is: * <0.05 and ** < 0.01. The exact p values obtained are indicated in the Material and Methods section. *Abbreviations*: AAV, AAV2/8.TBG.*hARSB*; ERT: monthly ERT.

Figure S2. Reduction of liver and kidney GUSB activity in mice receiving low doses of gene therapy and/or monthly ERT.

Figure S2

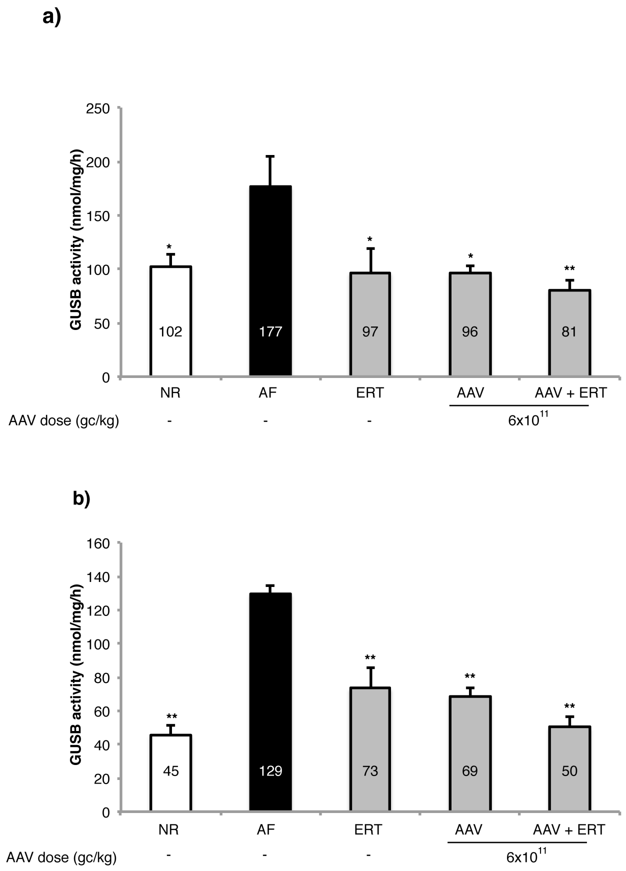

Beta-glucuronidase (GUSB) activity was measured in liver **(a)** and kidney **(b)** of treated MPS VI mice (gray bars), and of normal (NR, white bars) and affected (AF, black bars) controls. GUSB activity was averaged for all animals within the same group of treatment and the resulting value is reported as mean ± SE. The number of animals is 5 per each group. Statistical comparisons were made using the one-way ANOVA and the Tukey post hoc test. The *p* value vs AF is: *< 0.05 and **<< 0.01. The exact *p*-values obtained are indicated in the Material and Methods section. *Abbreviations*: AAV, AAV2/8.TBG.*hARSB*; ERT, monthly ERT.
